# Multiple incursions of foot-and-mouth disease virus serotype O into the Republic of Korea between 2010 and 2019

**DOI:** 10.1016/j.meegid.2024.105664

**Published:** 2024-10

**Authors:** Antonello Di Nardo, Da-Rae Lim, Soyoon Ryoo, Hyeonjeong Kang, Valerie Mioulet, Jemma Wadsworth, Nick J. Knowles, Jae-Myung Kim, Donald P. King, Sang-Ho Cha

**Affiliations:** a The Pirbright Institute, Ash Road, Pirbright, Woking, Surrey, United Kingdom; b Foot-and-Mouth Disease Research Division, Animal and Plant Quarantine Agency, Gimcheon-si, Republic of Korea

**Keywords:** Foot-and-mouth disease virus, Serotype O, Whole-genome sequencing, Molecular epidemiology, Outbreak reconstruction, Transmission tree, Republic of Korea, Asia

## Abstract

This study characterised type O foot-and-mouth disease (FMD) viruses recovered from outbreaks that were reported between 2010 and 2019 in the Republic of Korea. We used 96 newly generated whole-genome sequences (WGS) along with 131 already published WGSs from samples collected from countries in East and Southeast Asia. We identified at least eight independent introductions of O/SEA/Mya-98 and O/ME-SA/Ind-2001e FMDV strains into the Republic of Korea during the study period, which were closely related to the sequences of viruses circulating in the East and Southeast Asia neighbourhood with over 97 % nucleotide identity. Spatial-temporal transitions of O/SEA/Mya-98 lineage viruses recovered from the largest outbreak (2014–16) showed that after initial cases were detected within a 15-day period in July 2014, a single introduction of the same virus during December 2014 generated extensive forward virus transmission between farms that lasted until March 2016. We estimated that secondary transmissions were responsible for infection on 44 % FMD affected farms, over a total of 14 generations of infection. We eastimated a median evolutionry rate of 2.51 × 10^−5^ nt/site/day, which is similar for other FMD epidemic scenarios. These findings suggest that regular incursions of different FMDV lineages into the Republic of Korea have posed a continuous threat from endemic countries of East and Southeast Asia. These data highlight the importance of active cooperation and information exchange on FMD situation within Asian countries and assessment about the likely risk routes of virus movement is highly necessary to prevent further incursion and virus spread of FMDV in the Republic of Korea.

## Highlights

• Whole-genome sequences demonstrate distinct introductions of FMDV into South Korea.
• Viral sequences document at least eight incursions of FMDV serotype O (2010–2019).
• Analyses highlight risk pathways for FMDV introduction from neighbouring countries.
• Underreporting of FMD in Asia limit our ability to accurately define virus sources.

## 1 Introduction

Among the animal diseases of global concern, foot-and-mouth disease (FMD) has a major impact on livestock productivity and causes important economic consequences on trade of animals and animal products in countries where the FMD virus (FMDV), an *Aphthovirus* of the family *Picornaviridae*, is circulating (Knight-Jones and Rushton, 2013). In addition, the financial burden incurred by countries that have eradicated FMD is high if outbreaks reoccur due to a fresh introduction of the virus.

Eradication of FMD was achieved in the Republic of Korea (hereafter referred as South Korea) in 1935 and the country did not record any FMD outbreaks for more than 60 years. However, during the last 20 years, introduction of FMDV strains belonging to two serotypes circulating within the FMD endemic Pool 1, which includes East and Southeast Asia, have caused outbreaks on multiple occasions (Paton et al., 2009). The epidemiology of FMD in the neighbourhood has been characterised by the co-circulation of different FMDV strains belonging to serotype A (ASIA/Sea-97), O (ME-SA/PanAsia, ME-SA/PanAsia-2, SEA/Mya-98, ME-SA/Ind-2001 and CATHAY) and Asia 1 (Di Nardo et al., 2011; Valdazo-González et al., 2013; Brito et al., 2017). The first outbreaks were reported in South Korea in March 2000 and also during May–June 2002 (Shin et al., 2003) and caused by the O/ ME-SA/PanAsia FMDV lineage. Both of these events were controlled by a combination of culling of infected animals and use of a suppressive vaccination policy (vaccinate-to-kill), which allowed the country to regain the WOAH official status of free from FMD without vaccination by the end of 2002 (Park et al., 2014a). However, in January and April 2010 new FMD outbreaks were reported which were caused by FMDV lineages from two different serotypes: the A/ASIA/Sea-97 and O/SEA/Mya-98, respectively (Park et al., 2013a, Park et al., 2013b, Park et al., 2014b). Viruses causing these incursions were phylogenetically related to viruses circulating in mainland Southeast Asia and, while A/ASIA/Sea-97 cases were reported only for a short period during January 2010, circulation of O/SEA/Mya-98 within South Korea led to outbreaks on 166 farms over a period of more than a year. The last case was reported in April 2011 and, in total, cases due to O/SEA/Mya-98 caused economic losses of about US$ 3124 million (Park et al., 2014a). Retrospective phylogenetic analyses found that O/SEA/Mya-98 viruses were introduced on at least in two occasions from different sources (Knowles et al., 2012; Park et al., 2014a). Despite an official nationwide vaccination campaign which started in September 2011 and included serotype O as well as serotypes A and Asia1 vaccine strains, new cases to O/SEA/Mya-98 were reported again during July 2014 in Gyeongsangbuk-do and Gyeongsangnam-do. Although this was a minor event with only three pig farms declared as being infected (Park et al., 2014a), the same FMDV strain was reintroduced again in December 2014, resulting in infection on 185 farms over a 5-month period with the last case confirmed in April 2015 (Park et al., 2016, Park et al., 2018). Subsequently, from January to March 2016, 21 farms in the west of the country (Jeollabuk-do and Chungcheongnam-do) were reported to be infected by the same O/SEA/Mya-98 that was circulating during 2014–15, but no clear evidence was found to attribute the origin of these cases (Park, 2016).

More recently, the spread and expansion of the O/ME-SA/Ind-2001e FMDV strain within East and Southeast Asia has represented a major threat for the South Korean livestock industry (Bachanek-Bankowska et al., 2018). In February 2017, the first case due to this FMDV sublineage was reported on a farm in Boeun-gun, Chungcheongbuk-do (Lee et al., 2020). During the same time, a new introduction of the A/ASIA/Sea-97 strain was also detected in Gyeonggi-do, after a 7-year gap from the last occurrence of a serotype A in South Korea (Park, 2017). From March to April 2018, new outbreaks due to A/ASIA/Sea-97 FMDV lineage were reported in Gimpo-si (Gyeonggi-do) (Park, 2018). Subsequently in January 2019, the O/ME-SA/Ind-2001e strain was again confirmed infecting cattle farms in both Gyeonggi-do and Chungcheongbuk-do. The two O/ME-SA/Ind-2001 events were promptly controlled within a month of their initial onset and cases were only limited to few farms (8 in 2017 and 3 in 2019) (Upadhyaya et al., 2021). However, new cases due to this FMDV strain were recently reported during May 2023 in two counties (Cheongju and Jeungpyeong) of Chungcheongbuk-do (Ryoo et al., 2024), highlighting how the endemic circulation of FMDV lineages in the neighbouring region poses a continuous threat for South Korea, despite the on-going nationwide vaccination program.

In this paper we used 96 FMDV whole-genome sequences (WGSs) generated from isolates derived from clinical cases to reconstruct the regional context of the type O FMDV lineages that have caused outbreaks in South Korea between 2010 and 2019 (Table 1), namely the O/SEA/Mya-98 and O/ME-SA/Ind-2001e strains. These data were analysed together with 131 representative WGSs from contemporary viruses circulating in East and Southeast Asia in order to identify likely risk pathways of FMD virus introduction into South Korea and also reconstruct in more detail the space-time transitions of virus movements between farms that were infected by O/SEA/Mya-98 during 2014–16.

**Table 1:** Type O FMDV outbreaks reported in the Republic of Korea between 2010 and 2019. The most closely related FMDV WGSs for each outbreak are defined as those returning the highest percentage of nucleotide sequence identity (%nt identity).

Time	FMDV Lineage	No IP	Most-Closely Related Viruses [%nt identity]
Apr-2010	O/SEA/Mya-98	13	O/33-P/CHA/2010 [99.27 %] O/VN/LC169/2009 [99.16 %]
Nov-2010 to Apr-2011	O/SEA/Mya-98	153	O/RUS/Jul/2010 [99.24 %]
Jul-2014	O/SEA/Mya-98	3	O/HKN/20/2010 [96.45 %] O/JX/China/2010 [96.35 %]
Dec-2014 to Apr-2015	O/SEA/Mya-98	185	O/VN1/2014 [97.72 %]
Jan-Mar 2016	O/SEA/Mya-98	21	O/SKR/HS/139/2015 [99.28 %] O/SKR/HS/155/2015 [99.24 %] O/SKR/HS/150/2015 [99.23 %]
Feb-2017	O/ME-SA/Ind-2001e	8	O/XJ/CHA/2017 [99.47 %] Zabaikalskiy/1/RUS/2016 [99.40 %] O/MOG/BU/2–7/2015 [99.28 %]
Jan-2019	O/ME-SA/Ind-2001e	3	O/MYA/Yan/5/2016 [97.83 %] O/VIT/8338/2017 [97.25 %]

## 2 Materials and methods

### 2.1 Sequence data

FMDV WGSs were generated from 96 clinical samples collected from farms affected by FMD and officially reported by South Korea during 2010–11 (*n* = 8), 2014–16 (*n* = 80), 2017 (*n* = 3) and 2019 (*n* = 5) (Supplementary Table 1). WGSs of FMDV isolates were determined both at the World Reference Laboratory for FMD (WRLFMD), The Pirbright Institute – UK, and the FMD WOAH Reference Laboratory, Animal and Plant Quarantine Agency – South Korea, using Illumina MiSeq technology following a previously described NGS method (Logan et al., 2014).

Nucleotide sequence alignments were constructed using MAFFT 7.522 (Katoh and Standley, 2013). A further 131 FMDV WGSs generated from samples collected from countries in East and Southeast Asia were retrieved from the WRLFMD sequence database (www.fmdbase.org), which also includes FMDV genomes sourced from GenBank (Supplementary Table 1). We investigated presence of recombinant regions within the alignment by running the Genetic Algorithm for Recombination Detection (GARD) (Kosakovsky Pond et al., 2006) in HyPhy 2.5.59 (Kosakovsky Pond et al., 2020).

### 2.2 Phylogenetic inference

Model selection analysis and maximum-likelihood (ML) tree reconstruction were run in IQ-TREE 2.2.5 (Kalyaanamoorthy et al., 2017; Minh et al., 2020) using 1000 ultrafast bootstrap replicates (UFBoot) and 1000 SH-like approximate likelihood ratio tests (aLRTs) (Hoang et al., 2018). The general time reversible model with gamma-discretised among-site rate variation (GTR + Γ_4_) (Tavaré, 1986) was identified as the best-fitting nucleotide substitution model according to the computed Bayesian Information Criterion (BIC) scores and then used for subsequent analyses. The degree of temporal signal present in the sequence data was then evaluated using a root-to-tip regression of genetic distances against sampling time (Rambaut et al., 2016), computed using the ML tree. Temporally resolved phylogenies were reconstructed using BEAST 1.10.5 pre-release (Suchard et al., 2018). The evolution of FMDV was modelled by parameterising the process of nucleotide substitution using the GTR + Γ_4_ model, by allowing evolutionary rates to vary across branches according to a log-normal distributed relaxed molecular clock (Drummond et al., 2006), and by using the non-parametric Skygrid coalescent population prior with 100 transition points (Gill et al., 2013). The posterior estimates were obtained running a Markov chain Monte Carlo (MCMC) for 200 million iterations and recovering samples every 20,000 states. Mixing and convergence of the MCMC chain was assessed using Tracer 1.7.3 (Rambaut et al., 2018), ensuring sufficient sampling was achieved (where the Effective Sample Size returned values of at least 200 and not lower than 500 for each of the posterior parameters). The Maximum Clade Credibility (MCC) tree was generated by TreeAnnotator from 9000 posterior trees after removing the initial 10 % of sampled trees as burnin.

### 2.3 Evolutionary analysis of spatiotemporal dynamics

The relaxed-random walk (RRW) diffusion model (Lemey et al., 2010) implemented in BEAST was further performed to reconstruct the dispersal of FMD viruses in a continuous space for the type O SEA/Mya-98 outbreak reported in South Korea during 2014–2016. Geographic coordinates were extracted from the exact location of the farm from where clinical samples were collected and from which a sequence was generated. The Cauchy distribution was used to account for branch-specific variation in spatial dispersal rates. Substitution, molecular clock and coalescent models with their prior specifications were set identically to the initial evolutionary analysis defined above. The joint posterior estimates were obtained running an MCMC chain of 200 million of iterations, recovering samples every 20,000 states. Inferences were based on the resulting 9000 trees after discarding the initial 10 % of trees as burnin. Weighted dispersal velocity $v_{w}$ and diffusion coefficient $D_{w}$ of virus dispersal (Pybus et al., 2012) were estimated using *seraphim* (Dellicour et al., 2016) by analysing a subset of 100 trees uniformly sampled from the posterior set of reconstructed phylogenies.

### 2.4 Transmission network reconstruction

Auxiliary to the spatiotemporal phylodynamics reconstruction, inference of the transmission network resulting from the 2014–2016 South Korean FMD outbreak was performed using a Bayesian framework implemented in the R package *outbreaker2* (Campbell et al., 2018). The required prior distributions for the serial interval ω and latent period f were set using gamma distributions. For ω scale and shape parameters values were assigned as 8.16 and 0.88, respectively, which characterise a distribution of mean 7.1 days and a variance value of 53.2. For f scale and shape parameters were set as 7.22 and 0.5, respectively, defining a distribution of mean 3.9 days and variance 29.6. An MCMC chain of 1 million iterations was run for each dataset with a thinning frequency of 10,000. The transmission tree was reconstructed using the posterior set of 9000 trees resulting after discarding 10 % of the MCMC chain as burnin. Statistical and structural properties of the reconstructed transmission networks were examined using the *igraph* package for R (Csárdi et al., 2023).

### 2.5 Statistical analysis

Post-processing of generated results, statistical analyses and data visualisation were performed in R 4.3.2 (R Core Team, 2023), using the R packages *ggtree* (Yu et al., 2017), *ggplot2* (Wickham, 2016), *tweenr* (Pedersen, 2022a) and *ggraph* (Pedersen, 2022b).

## 3 Results

**Fig. 1:**
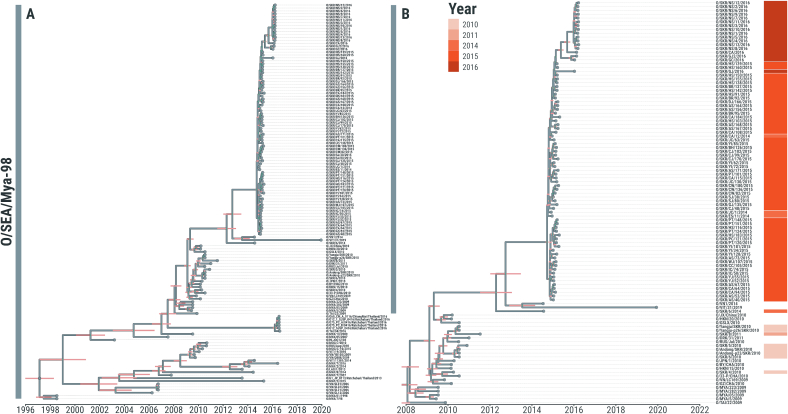
Evolutionary history of type O SEA/Mya-98 FMDV lineage incursions into the Republic of Korea between 2010 and 2016. (A) Time-stamped Maximum Clade Credibility (MCC) trees reconstructed using *n* = 227 FMDV WGSs generated from both O/SEA/Mya-98 (*n* = 135) and O/ME-SA/Ind-2001 (*n* = 92) isolates recovered from FMD outbreaks reported from countries in East and Southeast Asia. (B) Subtree of the O/SEA/Mya-98 phylogeny containing South Korean sequences collected during the 2010–11 and 2014–16 FMD outbreaks (*n* = 88). 95 % High Posterior Density (HPD) intervals for timing of node ancestry are represented by pink bars. Tips are coloured according to the year of sampling. Internal nodes with posterior support of >0.75 are coloured in cyan. (For interpretation of the references to colour in this figure legend, the reader is referred to the web version of this article.)

**Fig. 2:**
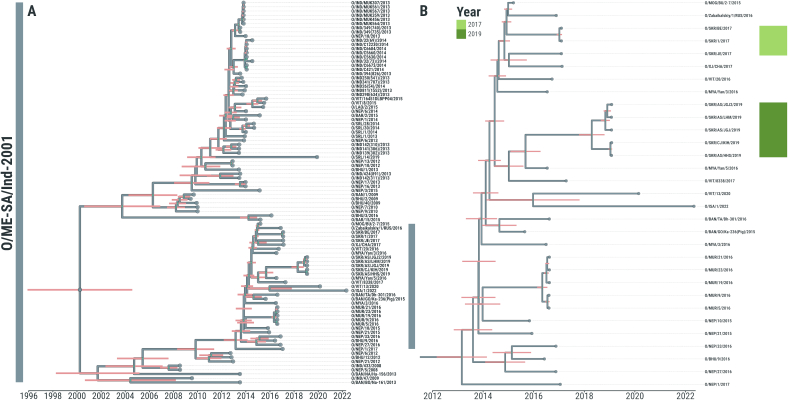
Evolutionary history of type O ME-SA/Ind-2001 FMDV lineage incursions into the Republic of Korea between 2017 and 2019. (A) Time-stamped Maximum Clade Credibility (MCC) trees reconstructed using *n* = 227 FMDV WGSs generated from both O/SEA/Mya-98 (*n* = 135) and O/ME-SA/Ind-2001 (*n* = 92) isolates recovered from FMD outbreaks reported from countries of the Eastern and South-eastern Asia regions. (B) Subtree of the O/ME-SA/Ind-2001e phylogeny containing Korean sequences collected during the 2017 and 2019 FMD outbreaks (*n* = 8). 95 % High Posterior Density (HPD) intervals for timing of node ancestry are represented by pink bars. Tips are coloured according to the year of sampling. Internal nodes with posterior support of >0.75 are coloured in cyan. (For interpretation of the references to colour in this figure legend, the reader is referred to the web version of this article.)

To investigate potential sources and likely routes of introduction of the O/SEA/Mya-98 and O/ME-SA/Ind-2001e FMDV lineages into South Korea during the last 10 years, we analysed 96 new WGSs generated from FMD clinical cases reported in the country between 2010 and 2019. These sequence data were combined with a dataset of further 131 WGSs, which included FMDV isolates collected from neighbouring regions. Of the publicly available sequences, 13.0 % were sampled from East Asia, 32.8 % from Southeast Asia and 52.7 % from Southern Asia, and in addition, two sequences were generated from FMD cases reported in Bahrain. Time-structured trees were constructed to explore the phylogenetic relationship of these FMDV strains, identifying 8 (95 % BCI 7–9) independent introductions of these serotype O viruses into South Korea during the last 10 years (Fig. 1, Fig. 2).

### 3.1 Reconstructing routes of introduction for South Korean type O FMDV strains

#### 3.1.1 Type O SEA/Mya-98 FMDV outbreaks occurring between 2010 and 2016

The O/SEA/Mya-98 FMD viruses causing outbreaks reported during 2010–11 were found to be most closely related to contemporary viruses isolated from East Asia (98.81 ± 0.17 % nucleotide identity) including China, Japan, North Korea and the Far Eastern Federal District of Russia (Zabaykalsky Krai). WGSs generated from clinical samples collected during the 2010–11 outbreaks were assigned within the Bayesian tree into three different clades, supporting the independent introductions of distinct viruses into South Korea at different times (Fig. 1B). Their most-recent common ancestors (MRCAs) were estimated to have been circulating on or around Sep-2009 (95 % BCI Jul- to Dec-2009). One of the viruses sampled during the 2010 outbreak was phylogenetically linked downstream and genetically similar to the O/SEA/Mya-98 isolate collected during 2011 (99.66 ± 0.07 % nucleotide identity), revealing how this introduction was associated to a more sustained transmission and further virus spread within the country.

We identified that all the O/SEA/Mya-98 FMD viruses isolated in South Korea during the outbreaks reported between Jul-2014 and Mar-2016 formed a single large monophyletic clade on a long branch (Fig. 1B), for which the MRCA was estimated to have been circulating during Dec-2011 (95 % BCI Jun-2011 to Jan-2013). The earliest sequenced FMDV isolate (O/SKR/6/2014) sampled during Jul-2014 from Uiseong-gun, Gyeongsangbuk-do, was assigned as singleton and basal to the clade, suggesting that this virus arose from an initial and distinct introduction that pre-dated the large series of FMD outbreaks that started in December 2014 (Fig. 1B). An FMDV isolate collected from Vietnam in 2014 was assigned into a further clade basal to the downstream core 2014–16 Korean FMD outbreak clade, which represent its most closely related sampled strain from the neighbouring region (97.70 ± 0.04 % nucleotide identity). More divergent sequences (96.25 ± 0.08 % nucleotide identity) of O/SEA/Mya-98 viruses collected during 2010 from China, including Hong Kong SAR, clustered as an earlier outgroup (Fig. 1B).

Phylogenetic assignment of the 2014–16 O/SEA/Mya-98 WGSs from South Korea suggests that the FMD outbreak event reported in December 2014 was caused by a single virus introduction that generated an extensive forward virus transmission (Fig. 1B), with its MRCA dated at Aug-2014 (95 % BCI Aug-2014 to Oct-2014). There was no evidence that this ancestral virus was circulating in South Korea before the first infection was detected. Further evidence for the sustained virus spread within-country was provided by the close phylogenetic relationship of FMDV cases reported during late Mar-2015 from the Chungcheongnam-do with FMDV isolates recovered one year later in Jan-Mar 2016 (99.17 ± 0.02 % sequence identity) (Fig. 1B). The MRCA of this later phase of the 2014–16 outbreak was estimated at Apr-2015 (95 % BCI Apr-2015 to Jul-2015).

#### 3.1.2 Type O ME-SA/Ind-2001e FMDV outbreaks occurring between 2017 and 2019

FMDV isolates associated with the first type O/ME-SA/Ind-2001e outbreak recorded in South Korea during 2017 were phylogenetically assigned into two sister clades (Fig. 2B), for which the MRCA was dated at Aug-2014 (95 % BCI Mar-2014 to Jan-2015). This topological reconstruction suggests that different viruses were independently introduced into South Korea on two occasions: FMDV isolate O/SKR/BE/2017 was found to be most closely related (99.28 ± 0.13 % sequence identity) with viruses circulating during 2015 and 2016 in the westernmost province of Mongolia (Bayan-Ölgii) and the Far Eastern Federal District of Russia (Zabaykalsky Krai), respectively (Fig. 2B); while O/SKR/JE/2017 was closely related to a different O/Ind-2001e virus isolated from the Xinjiang Uygur Autonomous Region of China in 2017 (99.47 % sequence identity) (Fig. 2B). Earlier FMDV sequences from mainland Southeast Asia (Myanmar and Vietnam) collected during 2016 were phylogenetically basal to the main clade. The WGSs generated from samples collected during the O/Ind-2001e outbreak reported from South Korea in January 2019 clustered in a monophyletic clade but split into two sister groups. These two groups of isolates were characterised by nucleotide differences of 46 ± 1.5 changes in their composition, despite being collected within a 4 day-period. Their MRCA was estimated around Jun-2018 (95 % BCI Sep-2017 to Nov-2018). However, no clear route of introduction could be assigned, since FMDV viruses isolated from neighbouring countries were not contemporary to the isolates recovered from the 2019 outbreak: the most closely related viruses (97.50 ± 0.31 % sequence identity) were identified in some temporally distant FMDV isolates collected from Myanmar and Vietnam during 2016 and 2017, respectively (Fig. 2B).

There was no evidence from the phylogenetic assignment that any of the South Korean FMD viruses had spread beyond the country.

#### 3.1.3 Molecular evolution of type O SEA/Mya-98 and ME-SA/Ind-2001e FMDV lineages

Molecular clock analyses estimated that the O/SEA/Mya-98 and O/ME-SA/Ind-2001 FMDV lineages evolved at a rate of 6.74 × 10^−3^ nt/site/year (95 % BCI 5.75 × 10^−3^–7.80 × 10^−3^) and 4.46 × 10^−3^ nt/site/year (95 % BCI 3.32 × 10^−3^–5.65 × 10^−3^), respectively. Dissection of the O/ME-SA/Ind-2001 phylogeny into the O/Ind-2001d and O/Ind-2001e monophyletic subtrees, provided estimates for the rate of FMDV evolution for each sublineage at 4.35 × 10^−3^ nt/site/year (95 % BCI 2.66 × 10^−3^–6.74 × 10^−3^) and 4.61 × 10^−3^ nt/site/year (95 % BCI 1.28 × 10^−3^–8.29 × 10^−3^), respectively.

Recombination analysis identified one topological breakpoint located within the 2B coding region (nt position 4055). On inspection of the maximum-likelihood phylogenies reconstructed by splitting alignment into the two genome fragments identified, no evidence of inter-serotypic recombination was found, with evidence of intra-serotypic recombinants confirming results already reported for the O/ME-SA/Ind-2001 lineage (Bachanek-Bankowska et al., 2018). However, the phylogenetic assignment of the South Korean isolates at the within-lineage level was largely conserved across the different genome fragments (Supplementary Fig. 2).

Park et al. (2016) reported the occurrence of a O/SEA/Mya-98 virus (KY322674) from South Korea which had a 23 amino acid deletion in the 3 A/3B1 region (effectively a deletion of VPg1) resulting in a reduced pathogenicity. In this study we have identified another O/Mya-98 virus (MAY/7/2016; PP916196) with a similar deletion. Additionally, TAI/34/2016 (PP916197), also an O/SEA/Mya-98 virus, has a mutation in 3B1 nucleotide position 7 (U to C) which results in an amino acid substitution of the critical RNA-attachment tyrosine to histidine, presumably leading to VPg1 being unable to function. This lack of function could lead to the eventual deletion of the 3B1 region. The distant relationships between the 3 A regions of these three viruses (87–91 % nt identity) suggests that these deletions/mutations occurred independently.

### 3.2 Dynamics of virus diffusion during 2014–16 for O/SEA/Mya-98

By combining all the epidemiological and genetic data available for 80 O/SEA/Mya-98 WGSs collected during 2014–16 from South Korea, the within-country dynamics of FMDV was reconstructed by analysing the network of virus transmission between farms. The total number of farms declared to be infected was 209; comprising three infected during the initial Jul-Apr 2014 event, 185 from Dec-2014 to Apr-2015 and 21 recorded for the latest phase during Jan-Mar 2016, respectively.

**Fig. 3:**
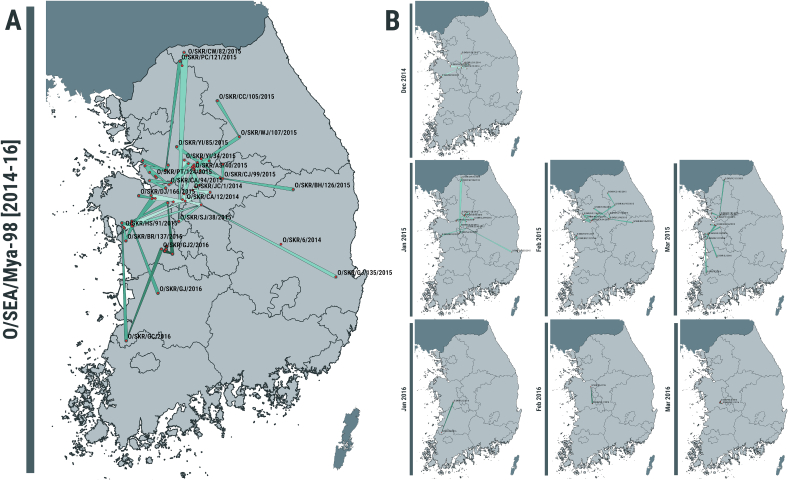
Spatio-temporal dynamics of the type O SEA/Mya-98 FMDV outbreaks reported by the Republic of Korea between 2014 and 2016. (A) Transmission tree reconstructed for the full outbreak time-window and mapped in geographical space. (B) Transmission links mapped in geographical space for each reporting month of the outbreak by extracting subtrees from the full transmission network. Directionality of the transmission links between infected farms inferred from the transmission tree analysis is expressed from the thin to the thick end of the line. Lines are coloured according to the time of reporting, expressed in months.

**Fig. 4:**
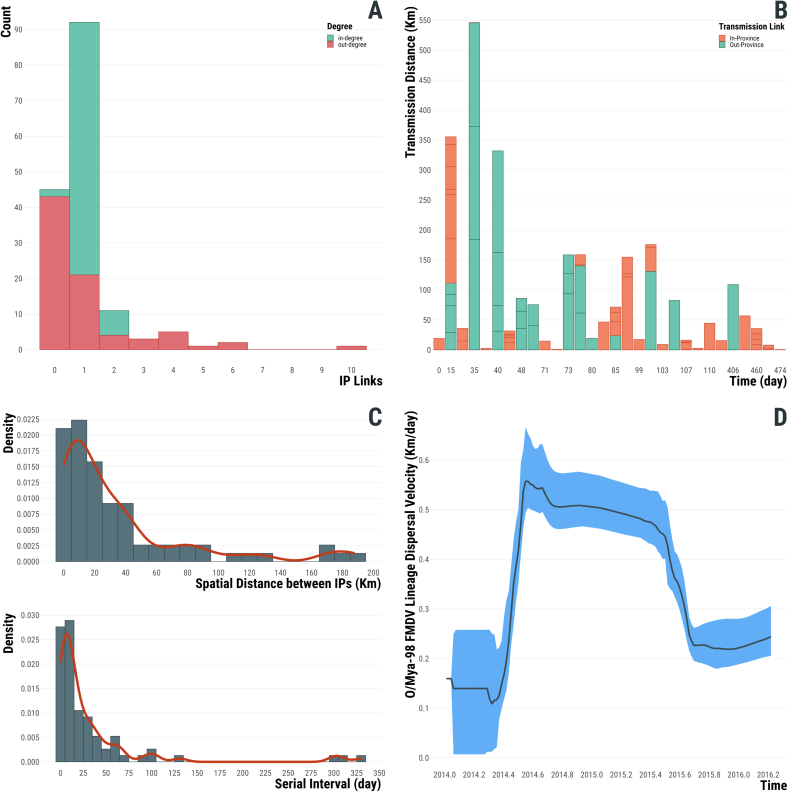
Epidemiological features of the type O SEA/Mya-98 FMDV outbreak reported between 2014 and 2016 in the Republic of Korea. (A) Transmission network connectivity identified through the measure of the degree centrality and categorised as in-degree (green) and out-degree (red) centrality measures. (B) Transmission distance (Km) through time (day) of the reconstructed epidemiological links and categorised according to virus movements either within (orange) or between (green) provinces. (C) [upper panel] Distribution of the geographical distance (Km) of the reconstructed epidemiological links between IP; [lower panel] distribution of the serial interval expressed in days. (D) Evolution of the weighted lineage dispersal velocity $v_{w}$ (km/day) through time estimated from a subsampled set of 1000 posterior trees with coloured areas representing the 95 % BCI (Bayesian Credible Interval). (For interpretation of the references to colour in this figure legend, the reader is referred to the web version of this article.)

**Supplementary Fig. 1:**
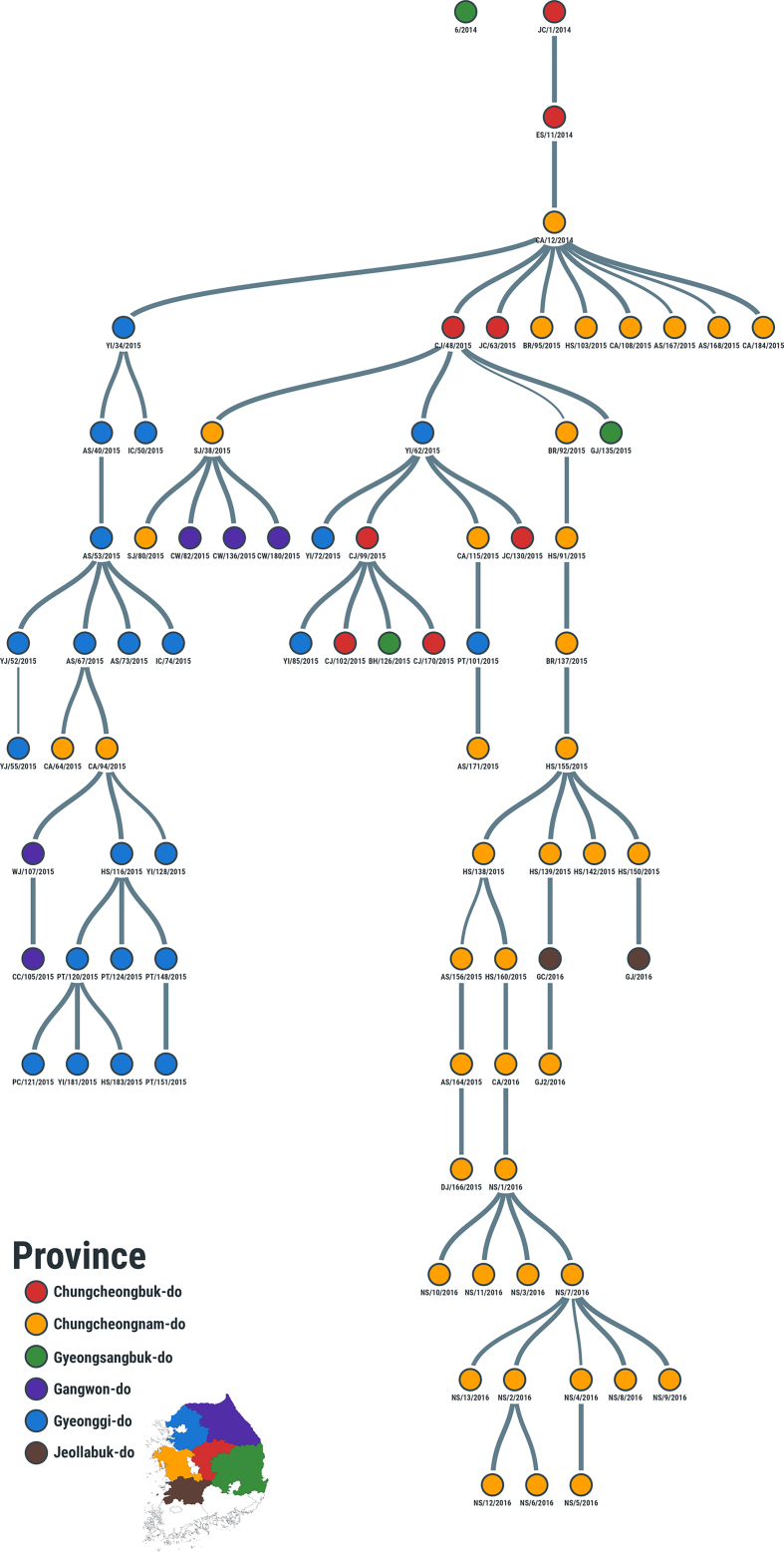
Transmission tree network of the type O SEA/Mya-98 FMD virus outbreak reported in the Republic of Korea between 2014 and 2016. The network is visualised according to the hierarchical structure of node connectivity. Colours of nodes define the South Korean province from where FMDV samples were collected, and as mapped in the inset. Edge width is proportional to ancestral support of the reconstructed link.

As already observed by phylogenetic analysis, the transmission tree generated using the 2014–16 data did not provide evidence of forward transmission linking the Jul-2014 outbreaks to the subsequent cases reported during Dec-2014 and in Apr-2015, thus designating them as two independent events (Fig. 3, Supplementary Fig. 1). This is revealed by estimating the giant component of the network, which contained 79 farms with the O/SKR/6/2014 FMDV isolate resulting the only unconnected node (Supplementary Fig. 1). The FMDV isolate (O/SKR/JC/1/2014) generated from a clinical case reported the 3rd of December 2014 from a pig farm in Jincheon-gun (Chungcheongbuk-do) was resolved as the index case of the 2014–16 outbreak with high probability (PP = 1) (Fig. 3B, Supplementary Fig. 1). Secondary transmissions were generated by 44 % (35/79) of the connected farms, with 12 farms characterised having a reproduction number $R_{t}\geq 2$ (Fig. 4A) and, among those, $R_{t}\geq 4$ was estimated for 8 farms. The serial interval distribution has a median of 12 days (95 % HDI 0–131) but characterised by a very long tail (median of 303 days) connecting the last cases in 2015 with the ones later discovered in early 2016 (Fig. 4C). The diameter of the transmission network revealed a total of 14 generations of infection along the transmission chain, whilst the average path length was estimated in 4.7 generations (Supplementary Fig. 1). Along the network diameter path, nt changes accruing from each transmission link was estimated in a median value of 12.5 (95 % PI 4.9–72.5), whilst during the full timeframe of the 2014–16 outbreak FMDV was found to evolve at a median rate of 2.51 × 10^−5^ nt/site/day (95 % BCI 2.16 × 10^−5^–2.86 × 10^−5^).

The estimated weighted dispersal velocity of 0.36 km/day (95 % BCI 0.32–0.42) is consistent with a relatively slow dispersal of the O/SEA/Mya-98 FMDV lineage during the timeline of the outbreaks where transmissions are predominantly at the local level (Fig. 4D). This tendency towards local transmissions was further supported by the distribution of geographical distances between pairs of connected farms (Fig. 4B), which had a median value of 18.6 km (95 % PI 0.2–174.6), and from which long distance transmission events were mainly mapped during the initial phase of the outbreak (between January and February 2015) (Fig. 3B). In addition, we identified a proportion of 0.66 reconstructed transmissions (50/76) that resulted in virus circulation within-province, whilst only a third of the transmission links (0.34, 26/76) accounted for virus movements between different provinces. Most of the FMDV transmissions reconstructed between provinces were traced from cases reported in Chungcheongnam-do (12/42), Chungcheongbuk-do (7/10) and Gyeonggi-do (6/22), whilst more than 70 % of infections within Chungcheongnam-do and Gyeonggi-do.

The proportion of cases sampled during the 2014–16 outbreak was estimated with a posterior median of 0.40 (95 % BCI 0.33–0.47), which indicates that ∼200 farms (95 % BCI 170–242) might have been infected during the entire time-window of the outbreak.

## 4 Discussion

Continued circulation of FMDV lineages belonging to different serotypes, coupled with an increasing mobility of people and goods, raise the risk of FMD transmission in the East Asia region (Knight-Jones and Rushton, 2013), despite the implementation of strict control policies and nationwide vaccination campaigns to prevent FMDV incursions. Therefore, a comprehensive knowledge of regional viral epidemiology is needed to identify risk routes of virus introductions and thus to better allocate resources. By using genomic analysis of WGSs generated from FMD viruses isolated from countries belonging to the FMDV endemic Pool 1, we reconstruct type O FMDV lineage incursions into South Korea reported during the last 10 years. Although official reports from the Korea government hint at seven independent incursions of type O FMDV viruses between 2010 and 2019 (MAFRA, 2023), the phylogenetic analyses performed in this study suggest that at least eight FMDV introductions have occurred during this time period. The phylogenetic placement of the FMDV sequences isolated during the type O SEA/Mya-98 outbreak reported during 2010, clearly indicates sequential introductions of different viruses into South Korea on three different occasions, and which were linked to viruses circulating in late 2009, early 2010 and late 2010 within countries of East Asia. No FMD vaccination was conducted in 2010, and no FMD outbreaks were reported between the 4th of June and the 28th of November 2010 due to the introduction of new viruses. Following the completion of a surveillance programme in which no further evidence of FMD was found in movement restricted areas, South Korea regained its official WOAH FMD-free status without vaccination (WOAH, 2023) until a new outbreak of type O occurred in late November 2010 (Park et al., 2014b). From our analyses, it is evident that viruses circulating from November 2010 were not related to those isolated during the outbreak reported in April 2010. However, considering the limited phylogenetic resolution presented by the poor FMD surveillance carried out within East and Southeast Asia during that period, it is difficult to prove whether the subsequent FMD outbreak reported in 2011 was either caused by a new independent virus introduction or a resurgence of infections due to sustained transmissions within-country. The latter is nevertheless more plausible given that the WGS generated from clinical cases sampled during 2011 is 99.7 % similar to those from late 2010 (100 % based on sequences of the VP1 coding region).

Following the extensive number of FMD outbreaks that caused significant economic losses during 2010–11, South Korea started a nationwide sero-surveillance and mandatory vaccination program (Park et al., 2021). Despite strict control strategies to prevent further FMDV incursions, infection due to O/SEA/Mya-98 FMDV was again reported in July 2014, causing the largest and longest FMD series of outbreak during this period. By combining epidemiological and genetic data to resolve the transmission network of FMD cases reported during the 2014–16 outbreak, we did not find any evidence of forward transmission linking the FMD cases identified within the 15-day period of July 2014 to subsequent cases reported during December 2014 and later in April 2015, thus designating them as two independent events. In addition, we showed that a single virus introduction causing the index FMD case of December 2014 (a pig farm in Jincheon-gun, Chungcheongbuk-do) generated an extensive forward virus transmission that sustained virus spread within South Korea, which lasted until March 2016. Our findings provide strong evidence for the resurgence of O/SEA/Mya-98 in South Korea in January 2016, ∼8 months after the last FMD cases reported during April 2015. As previously reported, new FMD infections reappearing in 2016 have been linked to poor biosafety measures and difficulties with the vaccination procedure undertaken on some farms (Ku et al., 2016). After the 2014–2016 outbreaks, post-vaccination monitoring was conducted to identify gaps in the vaccination program by estimating population immunity across different categories (Park et al., 2021). The genomic data analysed here is consistent with continuous virus evolution (likely due to undetected or silent infection), rather than to evolutionary dormancy of the virus (such as in fomites form) before the recrudescence of the disease and the appearance of new cases. Secondary transmissions were most likely caused by 44 % of the infected farms (35/79), whilst the outbreak was characterised by a relatively slow virus dispersal suggesting transmission happened at a local level. To confirm this point, we identified that a relatively high proportion of links comprised virus circulation within-provinces, whilst only approximately a third of the reconstructed links described virus movements between different provinces. From an evolutionary perspective, the rate of FMDV evolution for the 2014–16 O/SEA/Mya-98 South Korean outbreak (2.51 × 10^−5^ nt/site/day) is in line with what has been previously published in similar settings, by using WGS data generated from the FMD outbreaks in the UK in 1967, 2001 and 2007 (2.39 × 10^−5^, 2.37 × 10^−5^ and 2.51 × 10^−5^ nt/site/day, respectively) (Freimanis et al., 2016) and the FMD event reported in Japan during 2010 (1.95 × 10^−5^ nt/site/day) (Hayama et al., 2019). In addition, a similar evolutionary rate (2.18 × 10^−5^ nt/site/day) was obtained by estimating the molecular clock of the O/Ind-2001e FMDV outbreak reported in South Korea during 2019, by using the five WGSs analysed included in this study.

In 2017, outbreaks due to FMDVs belonging to both serotype O and A occurred simultaneously in South Korea. These serotype O outbreaks represented the first time that the O/ME-SA/Ind-2001e lineage was detected in Korea, affecting animals in Boeun-gun (Chungcheongbuk-do) and Jeongeup-si (Jeollabuk-do). Although these cases were separated by only one day, our analyses suggest that these were likely caused by two different variants of O/ME-SA/Ind-2001e. In fact, FMDV sequence variability observed between FMDV isolates recovered from both locations (63 nt changes in the WGS, 8 nt changes in the VP1 coding region) do not support a direct transmission between the two farms. Instead, the phylogenetic reconstruction indicates that these viruses were either independently introduced into South Korea likely through parallel routes or originated from the same place but one of them moved to an intermediate place then acquiring mutations before being finally introduced into the country. Three of the most-closely related FMD viruses from the neighbouring region were isolated from China (99.47 % sequence identity), Russia (99.40 % sequence identity) and Mongolia (99.28 % sequence identity), thus equally assigning one of these countries as potential source among others unknow locations. However, substantial underreporting of FMD cases from endemic countries in East and Southeast Asia, coupled with unstructured and opportunistic sampling of clinical cases, limits our ability to accurately identify sources of FMDV. This important analytical limitation is further evidenced by the phylogenetic placement of those FMDV isolates generated during the second O/ME-SA/Ind-2001e outbreak recorded in South Korea during 2019 for which no clear route of introduction could have been identified, given that some of these isolates differ on average by 46 nt and no WGSs from contemporary circulating viruses were generated within the FMD virus pool 1. However, genotyping reports generated by the FAO World Reference Laboratory for FMD using FMDV sequences encoding for the 1D/VP1 region (WRLFMD, 2019) found that the 2019 South Korean isolates share 99.5 % of nucleotide identity with a virus isolated from the Zunyi prefecture (Guizhou province) of China during June 2018 (O/GZZY/CHA/2018-B). These outbreaks reported in 2019 were controlled within four days by prompt recognition of cases and the use of well-established disease control measures. These policies restricted infections to only three cattle farms. However, in 2023, the same FMDV lineage occurred in South Korea again (Ryoo et al., 2024).

When performing molecular epidemiological study, a comprehensive analytical approach using both FMDV sequences encoding the VP1 region and the full-genome length should be taken. It is generally accepted that phylogenies based on WGS and VP1 coding sequence data show differences in their resolution and sometime in their topological structure and, therefore, the utilisation of either WGS, capsid or VP1 coding regions should be driven by the aim of the study, i.e. WGS analyses are anticipated to offer higher resolution than VP1 when the transmission network of an outbreak is needed to be resolved (Bachanek-Bankowska et al., 2018). The VP1 coding region tends to change by 1 % per year, while at the WGS-level 1 to 7 substitution can be observed during direct farm-to-farm transmission (Cottam et al., 2008; Valdazo-González et al., 2015). Therefore, when FMDV sequences are used to identify virus sources at the country level, each criterion can lead to a different outcome: an outbreak caused by a residual virus versus an incoming one. FMDV sequences generated from outbreaks reported in South Korea on April 2010 and November 2010 showed a VP1 genetic difference in the nt composition of 1.56 % (10/639), whilst WGS showed 2.64 % (221/8357) of nt difference. FMDV isolates recovered from the 2017 outbreak showed a maximum difference of 1.25 % (8/639) in the nt composition of the VP1 coding region, with a maximum of 65 nt changes (0.78 %) reported between WGSs. Similarly, the difference in the VP1 coding region of FMDV genomes generated using samples collected from the first and second farms affected by the 2019 outbreak was of 4.5 %, whilst by WGSs a maximum of 48 nt differences (0.57 %) were estimated between them, making it difficult to conclude that direct transmission occurred between those farms. The FMDV isolates recovered from the 2014–16 outbreak were found to differ by 3.44 % (22/639) from either O/SKR/1/2014 or O/SKR/6/2014 in VP1 analysis, but when analysing the WGS sequence 228 nt changes were estimated (2.72 %). This study showed the results of various phylogenetic analyses using WGSs with the explicit aim of resolving likely routes of FMDV incursion into South Korea, investigating at a higher resolution genomic level than a regional perspective.

## 5 Conclusion

This study documents regular incursions of different FMDV lineages into the South Korea with likely sources in endemic countries of East and Southeast Asia. Risk pathways that have been identified for virus incursions include migration of foreign workers, international travellers, imported hay, wildlife migrations, yellow dust or imported animal products (Park et al., 2014a). While illegal/unregulated movements of live animals represent an import risk for some East Asia countries, we believe that this route is not important for South Korea, given the strict maritime and land border control measures placed on custom entry into the country. By itself, the phylogenetic analyses reported in this study do not confirm or refute any of these risks, and active cooperation and further analyses on the FMD situation within Asian countries are required to pinpoint the specific risk routes that underpin virus movements and to develop policies and approaches to reduce further incursions of FMD into the Republic of Korea. Control of FMD in the Republic of Korea should therefore be based on the identification of those risk factors and farms at high-risk of infections (Lee et al., 2021), while assessing the most probable sources and likely routes of FMDV introduction by relying on accurate and comprehensive surveillance data.

The following are the supplementary data related to this article.

**Supplementary Fig. 2:**
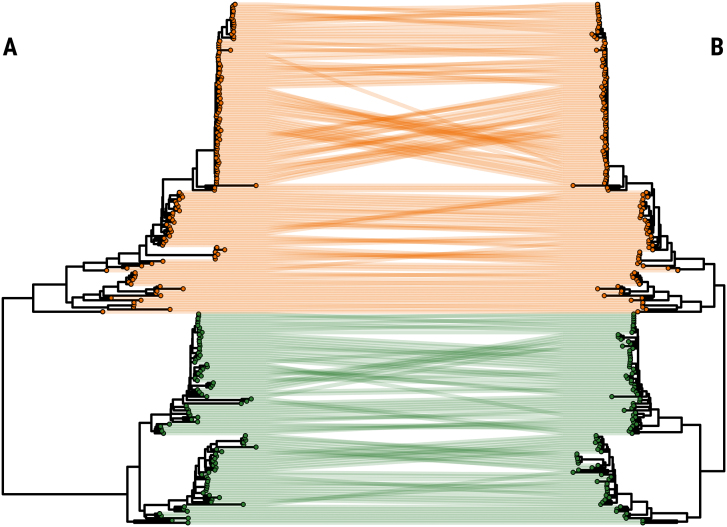
Tanglegram of phylogenies reconstructed for each individual fragment of the FMDV genome identified through recombination breakpoint analysis. Maximum-likelihood phylogenies reconstructed using the fragments of the FMDV genome including the 1-4056 (A) and 4057-8357 (B) nt sites, as estimated using GARD. Topological variation in virus isolates is denoted by interconnecting lines. Tips and lines are coloured according to the FMDV lineage; orange = O/SEA/Mya-98; green = O/ME-SA/Ind-2001.

Supplementary Table 1 Descriptive metadata of the n = 96 FMDV whole genome sequences and n = 131 reference sequences included in the study. Supplementary Table 1

## Funding

This research was supported by the Republic of Korea 10.13039/501100008771Animal and Plant Quarantine Agency research grant [grant number: B-1543082-2022-26-1]. Work at Pirbright was supported by the Department for Environment, Food, and Rural Affairs (Defra; UK) [grant number: SE2945] and funding provided by the 10.13039/501100000780European Union (via a contract from EuFMD, Rome, Italy). The views expressed herein can in no way be taken to reflect the official opinion of the European Union. The 10.13039/501100000870Pirbright Institute receives grant-aided support from the 10.13039/501100000268Biotechnology and Biological Sciences Research Council of the United Kingdom [grant numbers: BB/X011038/1; BB/X011046/1].

## CRediT authorship contribution statement

**Antonello Di Nardo:** Writing – review & editing, Writing – original draft, Visualization, Software, Methodology, Formal analysis, Data curation, Conceptualization. **Da-Rae Lim:** Writing – review & editing, Writing – original draft, Resources, Methodology, Data curation. **Soyoon Ryoo:** Supervision, Resources, Project administration, Funding acquisition. **Hyeonjeong Kang:** Validation, Resources. **Valerie Mioulet:** Validation, Resources, Methodology. **Jemma Wadsworth:** Validation, Resources, Methodology. **Nick J. Knowles:** Writing – review & editing, Validation, Resources, Methodology. **Jae-Myung Kim:** Supervision, Project administration, Funding acquisition. **Donald P. King:** Writing – review & editing, Supervision, Funding acquisition, Conceptualization. **Sang-Ho Cha:** Writing – review & editing, Supervision, Project administration, Funding acquisition, Conceptualization.

## Declaration of competing interest

The authors declare no competing interests.

## Data Availability

Newly generated FMDV WGSs (*n* = 96) that support the findings of this study have been deposited in GenBank. Accession numbers and associated metadata are listed in Table S1, which also provides information on previously deposited WGSs (*n* = 131).
